# GDF15 contributes to radioresistance and cancer stemness of head and neck cancer by regulating cellular reactive oxygen species via a SMAD-associated signaling pathway

**DOI:** 10.18632/oncotarget.13649

**Published:** 2016-11-26

**Authors:** Yan-Liang Li, Joseph T. Chang, Li-Yu Lee, Kang-Hsing Fan, Ya-Ching Lu, Yi-Chen Li, Chang-Hsu Chiang, Guo-Rung You, Hsin-Ying Chen, Ann-Joy Cheng

**Affiliations:** ^1^ Department of Medical Biotechnology, College of Medicine, Chang Gung University, Taoyuan 333, Taiwan; ^2^ Department of Radiation Oncology, Chang Gung Memorial Hospital, Taoyuan 333, Taiwan; ^3^ Department of Pathology, Chang Gung Memorial Hospital, Taoyuan 333, Taiwan

**Keywords:** GDF15, radioresistance, cancer stemness, reactive oxygen species (ROS), head and neck cancer (HNC)

## Abstract

Radiotherapy is an integral part for the treatment of head and neck cancer (HNC), while radioresistance is a major cause leads to treatment failure. GDF15, a member of the TGF-β superfamily, is hypothesized to participate in various types of homeostasis. However, the potential role of this molecule in regulation of radiosensitivity remains unclear. In this study, we demonstrated that GDF15 contributed to radioresistance of HNC, as determined by both gain- and lost-of-functional experiments. These results were achieved by the induction of mitochondrial membrane potential and suppression of intracellular reactive oxygen species (ROS). We further showed that GDF15 facilitated the conversion of cancer stemness, as assessed by the promotion of CD44+ and ALDH1+ cell populations and spheroid cell formation. At molecular level, GDF15 conferred to these cellular functions was through phosphorylated SMAD1 proteins to elite downstream signaling molecules. These cellular results were further confirmed in a tumor xenograft mouse study. Taken together, our results demonstrated that GDF15 contributed to radioresistance and cancer stemness by regulating cellular ROS levels via a SMAD-associated signaling pathway. GDF15 may serve as a prediction marker of radioresistance and a therapeutic target for the development of radio-sensitizing agents for the treatment of refractory HNC.

## INTRODUCTION

Head and neck cancer (HNC) is one of the most prevalent cancers worldwide [1–3]. The standard treatment for patients with HNC is surgery, radiation or chemotherapy or a combination of these treatments [2–4]. Although treatment strategies have advanced in the last two decades, the overall 5-year survival rate for patients has not significantly changed [3–5]. Local recurrence after radiotherapy is a major obstacle to recovery in HNC [3–5]. It is essential to elucidate the mechanisms underlying this disease to develop a more effective therapeutic modality. To obtain a more complete profile of the molecules that contribute to radioresistance, we previously established several sublines derived from HNC cell lines with highly radioresistant phenotypes [6, 7]. The cDNA microarray method was used for transcriptomic profiling of nasopharyngeal cancer cell lines [6], and comparative proteomic analysis was performed to search for potential proteins in oral cancer cells associated with radioresistance [7]. GDF15 was highly expressed in various radioresistant sublines by both screening methods. These results suggest that GDF15 may play a crucial role in the regulation of radio-sensitivity in HNC. However, the underlying mechanism by which this molecule leads to radioresistance remains unclear.

GDF15 (growth differentiation factor 15), also known as MIC-1, PLAB, P-TGFβ, PDF, and HP00269, was discovered and cloned simultaneously by separate groups [8, 9]. GDF15 is expressed as a 40 kDa propeptide that is cleaved in the endoplasmic reticulum to release a 25 kDa dimeric protein in circulation [8–10]. GDF15is a TGF-β/bone morphogenetic protein (BMP) super-family member that is expressed in a wide range of tissues, indicating that this molecule has broad biological activity [9–10]. Similar to other TGF-β family member, GDF15 exhibits cytokine activity and is induced by cellular stress conditions and responds to microenvironment stimulators [9–10]. Recently, several studies have reported that GDF15 is dysregulated in cancers. For examples, GDF15 possesses pro-tumorigenic functions, as indicated by the high expression or secretion in many types of cancer tissues, including colorectal, gastric, esophageal, oral, pancreatic, liver, and ovarian cancers [11–26]. This elevated expression was often associated with more aggressive diseases [22–25]. However, tumor suppressive effects have been found in breast, bladder, and endothelial cancer cells [26–28]. Apparently, GDF15 has a complicated role during tumorigenesis, which may be dependent on the specific tissues or disease conditions.

In the present study, we determined whether and how GDF15 contributes to radioresistance in HNC. We found that GDF15 contributes to radioresistance and facilitates cancer stemness conversion through regulation of cellular reactive oxygen species (ROS) via a SMAD-associated pathway. These findings were also supported by a xenograft mouse study, which demonstrated that tumor with higher GDF15 exhibited significant resistance to radiation treatment. GDF15 may serve as a predictive marker of radioresistance and a sensitizing target for the treatment of refractory HNC.

## RESULTS

### GDF15 contributes to radioresistance by suppressing cellular reactive oxygen species (ROS)

**Figure 1:**
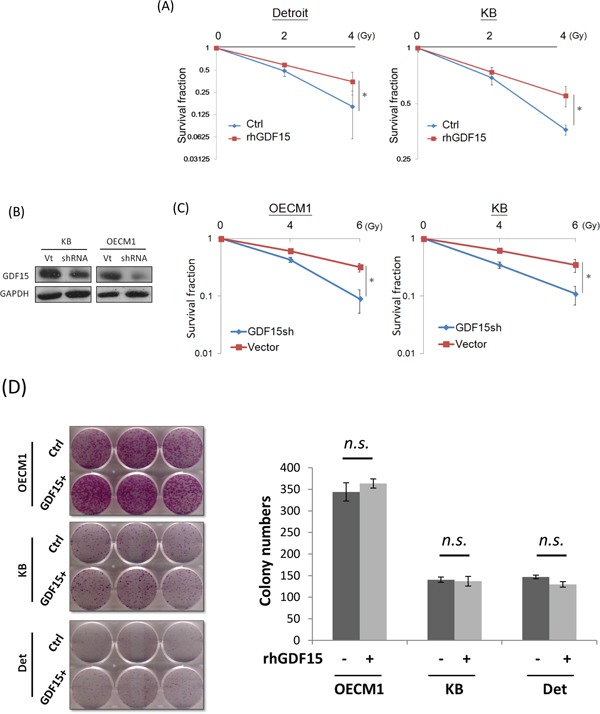
GDF15 contributes to cancer cell radioresistance but not cell growth **A.** Administration of rhGDF15 led to radioresistance in two HNC cell lines. Detroit and KB cells were treated with 20 ng/ml rhGDF15 for 5 days and then subjected to various doses of irradiation (0, 2, and 4 Gy). Fourteen days later, the cell colonies were determined by staining with 0.05% crystal violet (n=3). **B.** GDF15 expression was successfully inhibited after transfection of GDF15-specific shRNA plasmids (GDF15sh) in KB and OECM1 cell lines. After 48 h, cells were harvested for western blot analysis. **C.** Silencing GDF15 sensitized cells to irradiation in HNC cells. OECM1 and KB cells were transfected with GDF15sh or the vector plasmids and then subjected to irradiation (0 to 6 Gy). After 14 days, the cell colonies were determined (n=3). **D.** GDF15 had minimal effect on growth regulation in HNC cells. The OECM1, Detroit and KB cells were treated with or without rhGDF15 (20 ng/ml for 5 days). After 14 days, the cell colonies were determined (n=3). (*: *p* <0.05, *n.s.*: non-significance, *t*-test).

As GDF15 was found to be substantially overexpressed in various radioresistant sublines [6, 7], we confirmed whether GDF15 functionally contributes to radioresistance. In vitro gain-of-function experiments were performed by addition of recombinant human GDF15 (rhGDF15) protein to the culture medium. The potential effect on radiosensitivity was determined by clonogenic survival assays. The results are shown in Figure 1A. Administration of rhGDF15 significantly increased radioresistance in two HNC cell lines, with 1.3- to 1.7-fold increases in the number of surviving colonies at 4 Gy of irradiation. Loss-of-function experiments using GDF15-shRNA (GDF15sh) transfection were conducted. Silencing of GDF15 (Figure 1B) sensitized cells to irradiation, by reducing colony survival to 66-72% in two HNC cell lines (Figure 1C). Nevertheless, modulation of GDF15 had a minimal effect on cell growth in all tested cell lines (Figure 1D). These results suggested that GDF15 protects cells against radiation treatment and contributes to radioresistance.

**Figure 2:**
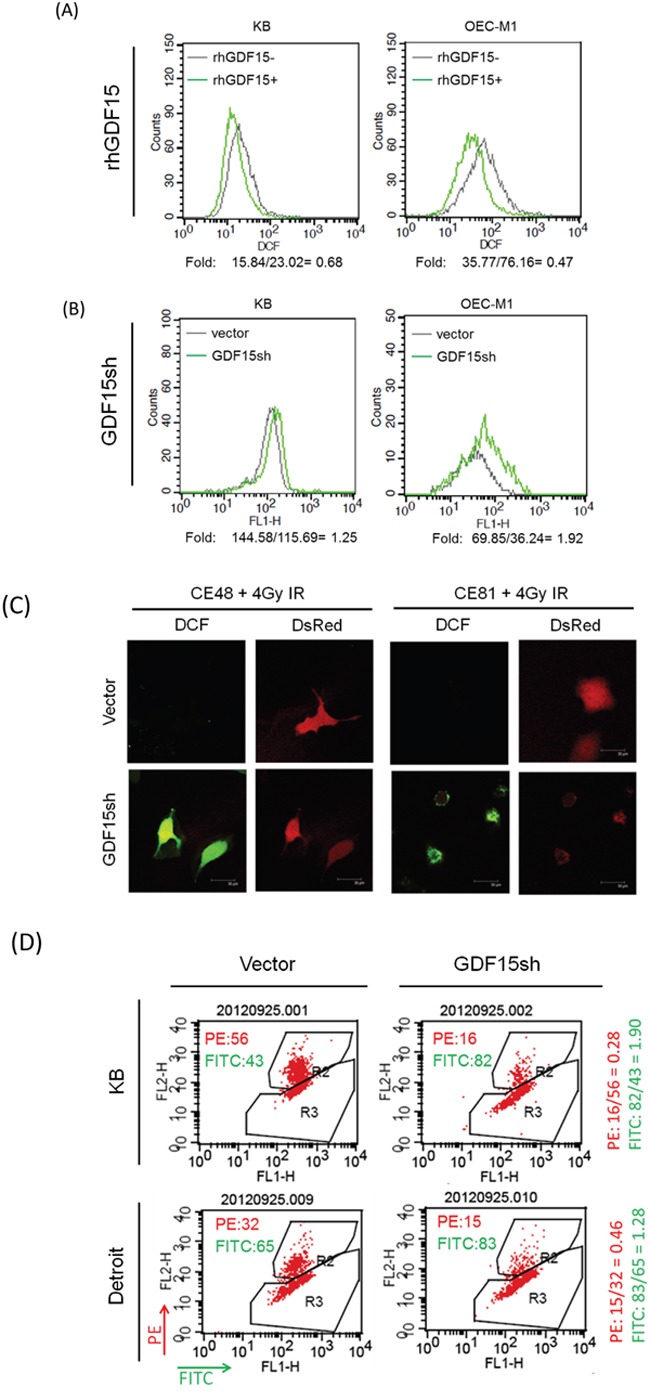
GDF15 suppresses the intracellular reactive oxygen species levels by altering the mitochondrial membrane potential **A.** Administration of rhGDF15 suppressed intracellular ROS level in HNC cells. KB and OECM1 cells were treated with 20 ng/ml of rhGDF15 for 5 days. After staining with DCF dye, the cells were subjected to flow cytometric analysis to determine the intracellular ROS levels. All values are presented as fluorescence intensity. **B.** GDF15 silencing increased ROS production in HNC cells. After transfection with GDF15sh or the vector plasmids, the KB and OECM1 cells were stained with DCF dye and subjected to flow cytometric analysis. **C.** GDF15 silencing increased ROS production in esophageal cancer cells. CE48T/VGH and CE81T/VGH cells were co-transfected with DsRed and GDF15sh/vector plasmids and then subjected to 4 Gy of irradiation. After staining with DCF dye, the cells were subjected to confocal microscopy. **D.** GDF15 silencing reduced mitochondrial membrane potential and led to apoptosis in HNC cells. KB or Detroit cells were transfected with the GDF15sh or the vector plasmids for 48 h. After incubating with MitoCapture reagents, the cells were subjected to flow cytometry analysis. The PE fluorescence fraction represents healthy cells, and the FITC fraction represents apoptotic cells.

It is well established that ionizing radiation can induce ROS in the cell, leading to apoptosis [29]. Therefore, we determined whether GDF15 promotes radioresistance through regulation of cellular ROS levels. The intracellular ROS were measured using the H_2_DCF-DA oxidation method, and the green fluorescence DCF product was analyzed by flow cytometry [7]. Administration of rhGDF15 reduced intracellular ROS levels to 47-68% in two HNC cell lines (Figure 2A). GDF15 silencing increased ROS production by 1.3- to 1.9-fold in HNC cells (Figure 2B). This phenomenon was observed in esophageal cancer cells as well. The radiation-induced ROS generation was substantially higher in GDF15-silenced cells as determined by the green fluorescence using DCF staining in two cell lines (Figure 2C). These data suggest that GDF15 has a role in the suppression of ROS generation. We further determined whether mitochondria, the most common source of ROS generation, were disturbed by GDF15. The MitoCapture assay was used to detect the disruption of mitochondrial membrane permeability, which may lead to cell apoptosis [30]. The kit utilizes a cationic dye that fluoresces differently in healthy (red fluorescence) and in apoptotic (green fluorescence) cells using flow cytometry. As shown in Figure 2D, GDF15 silencing increased the fraction of apoptotic cells (FITC population) by 1.3- to 1.9-fold in two HNC cell lines, suggesting that GDF15 knockdown decreased mitochondrial membrane potential and led to apoptosis.

Taken together, these results suggest that GDF15 promotes radioresistance through suppression of intracellular ROS production, which may involve the increase of mitochondria membrane potential. Silencing of GDF15 resulted in ROS generation, which led to induction of cell apoptosis and increased radiosensitivity.

### GDF15 promotes cancer stemness via facilitation of CD44+ and ALDH1+ cell populations

**Figure 3:**
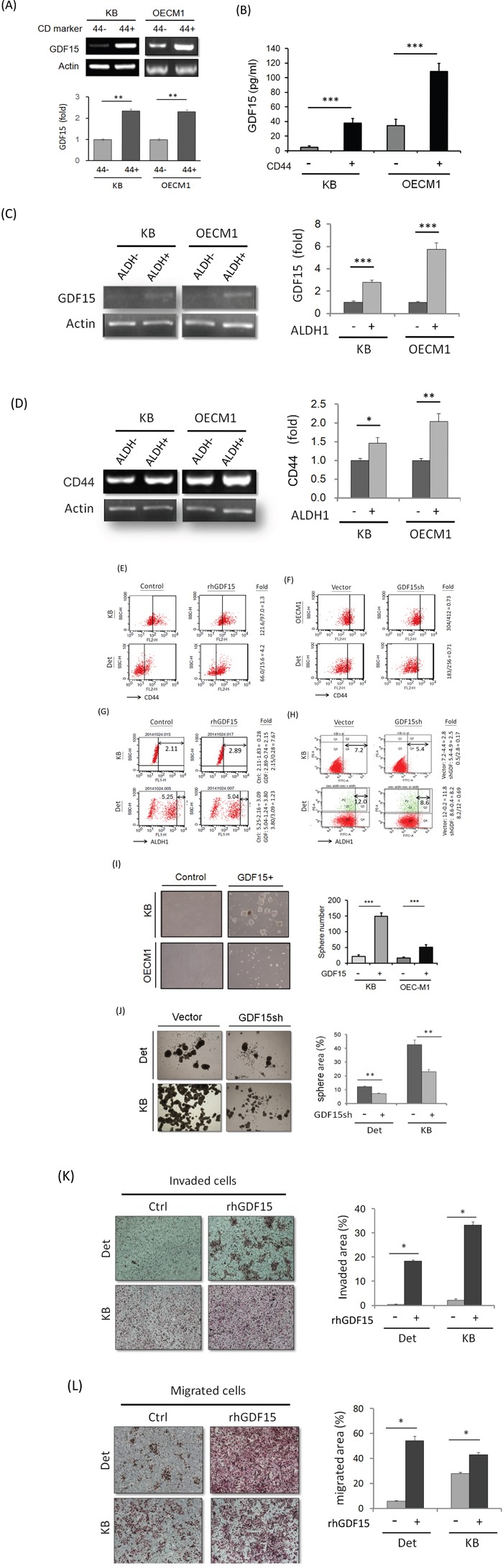
GDF15 promotes cancer stemness via facilitation of CD44+ and ALDH1+ cell populations **A.** Elevation of GDF15 mRNA expressions in CD44+ sorted cells compared to the CD44- cells in both KB and OECM1 cell lines, as determined by RT-PCR method (n=3). **B.** Elevation of GDF15 protein levels in the conditional medium of CD44+ sorted cells compared to the CD44- cells in both KB and OECM1 cell lines, as determined by ELISA method (n=3). **C.** Elevation of GDF15 mRNA expressions in ALDH1+ sorted cells compared to the ALDH1- cells in both KB and OECM1 cell lines, as determined by RT-PCR method (n=3). **D.** Elevation of CD44 mRNA expressions in ALDH1+ sorted cells compared to the ALDH1- cells in both KB and OECM1 cell lines, as determined by RT-PCR method (n=3). **E.** Elevation of CD44+ population in rhGDF15 treated cells compared to non-treated cells in both KB and Detroit cell lines, as determined by FACS method. **F.** Reduction of CD44+ population in GDF15sh transfected cells in both KB and Detroit cell lines, as determined by FACS method. **G.** Elevation of ALDH1+ population in rhGDF15 treated cells in both KB and Detroit cell lines, as determined by FACS method. **H.** Reduction of ALDH1+ population in GDF15sh transfected cells in both KB and Detroit cell lines, as determined by FACS method. **I.** Enhancement of spheroid cell formation in the GDF15+ sorted cells compared to GDF15- cells in both KB and OECM1 cell lines (n=3). **J.** Reduction of spheroid cell formation in GDF15sh transfected cells in both Detroit and KB cell lines (n=3). **K.** Increase of invasion ability in the rhGDF15 treated cells compared to non-treated cells (control) in both Detroit and KB cell lines (n=3). **L.** Increase of migration ability in the rhGDF15 treated cells compared to non-treated cells (control) in both Detroit and KB cell lines (n=3). (*: *p*<0.05, **: *p*<0.01, ***: *p*<0.001, *n.s.*: non-significance, *t*-test).

As radioresistance is a characteristic of cancer stem cells [31], we examined whether GDF15 also plays a role in cancer stemness. Because CD44+ and ALDH1+ have been characterized as markers of HNC stem cells [32, 33] we also determined the potential association between GDF15 levels and the CD44+- and ALDH1+-enriched populations. The CD44+ or ALDH1+ cell population was isolated by fluorescence-activated cell sorting (FACS) analysis, and the level of GDF15 expression was determined. GDF15 expression was increased in CD44+-enriched cells by 1.9- to 2.4-fold compared to the CD44- populations in two HNC cell lines (Figure 3A). Also, the secreted GDF15 was significantly higher in the conditional medium of the CD44+ cultured cells, with a 2.5- to 10-fold elevation as determined by ELISA (Figure 3B). For the ALDH+-enriched cells, GDF15 expression was increased by 2.8- to 5.7-fold compared to the ALDH1- populations in two HNC cell lines (Figure 3C). Concomitantly, these ALDH1+-enriched cells also showed higher CD44+ levels (Figure 3D). These results suggest that GDF15 expression was positively associated with the CD44+ and ALDH1+ populations in HNC.

We next examined whether the CD44+ or ALDH1+ cell populations can be regulated by GDF15 by modulating the GDF15 levels via rhGDF15 or GDF15sh transduction. Administration of rhGDF15 increased the CD44+ populations by 1.3- to 4.2-fold in two HNC cell lines (Figure 3E). Conversely, GDF15 silencing reduced the CD44+ population to approximately 70% in HNC cells (Figure 3F). For the ALDH+ population, administration of rhGDF15 increased the ALDH1+ cell population by 1.3 to 7.7-fold (Figure 3G), whereas GDF15 silencing decreased this population to 17-69% in two cell lines (Figure 3H). These results suggest that GDF15 facilitated the CD44+ and ALDH1+ cell populations.

As self-renewal is a typical property of cancer stemness [31–33], we assessed this cellular characteristic in GDF15+ cells. FACS was used to isolate the GDF15+ sub-population from HNC cell lines. The GDF15+ cells showed enhanced spheroid cell formation compared to the GDF15- cells, as evidenced by the increased sphere size and number (3- to 6-fold increase) (Figure 3I). GDF15 silencing significantly suppressed spheroid cell formation to 53-59% in two HNC cell lines (Figure 3J). Furthermore, this molecular silencing substantially inhibited cellular migration (Figure 3K) and invasion (Figure 3L), attributes of cancer stemness [31–33]. Taken together, these data suggested that GDF15 promoted cancer stemness conversion by facilitating CD44+ and ALDH1+ cell formation.

### GDF15 modulates cellular ROS levels to promote cancer stemness

**Figure 4:**
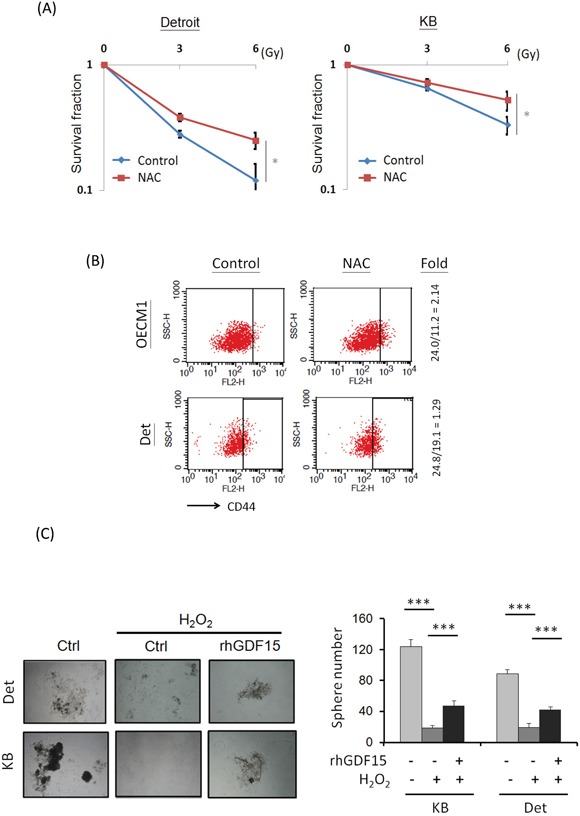
GDF15 modulates cellular ROS levels to promote cancer stemness **A.** Treatment of antioxidant agent led to radioresistance in HNC cells. After treatment of 10 μM N-acetylcysteine (NAC) for 48 h, Detroit or KB cells were subjected to serial dose of irradiation (0 to 6 Gy). Fourteen days later, the numbers of surviving cell colonies were determined (n=3). **B.** Treatment of antioxidant agent increase CD44+ cell population. After treatment of 10 μM NAC for 48 h, the OECM1 or KB cells were subjected to flow cytometry analysis for CD44+ populations. **C.** GDF15 reversed the effects of ROS in the suppression of spheroid cell formation. Detroit or KB cells were treated with H_2_O_2_ (5 μM) with or without addition of rhGDF15 (20 ng/ml) for 48 h. These cells were then incubated in the spheroid cell culture condition and assessed after 14 days (n=3). (*: *p* < 0.05, **: *p* < 0.01, ***: *p* < 0.001, *t*-test).

Because GDF15 modulates ROS generation (Figure 2), we examined whether the GDF15 contribution to cancer stemness is associated with ROS regulatory mechanisms. For this, an antioxidant agent, N-acetylcysteine (NAC), was used to reduce cellular ROS levels [35, 36]. The effect of NAC on radiosensitivity was first examined. As shown in Figure 4A, NAC treatment increased the surviving fraction of HNC cells by 1.6- to 2-fold after exposure to radiation at 6 Gy. To determine whether lower ROS levels could affect cancer stemness in HNC, cells were treated with NAC, and the CD44+ population was assessed by FACS. As shown in Figure 4B, the CD44+ population was increased after NAC treatment by 1.3- to 2.1-fold in two HNC cell lines. These results suggest that cancer stemness is associated with cellular ROS levels, and lower oxidative stress could enhance stemness.

We further examined whether the ROS-cancer stemness regulatory axis can be modulated by GDF15, by using spheroid cell formation assays. As shown in Figure 4C, spheroid cell formation was substantially inhibited by H_2_O_2_ treatment to 78-85% compared to the controls. However, this reduction was rescued following rhGDF15 administration, with an approximately 2-fold increase in the two HNC cell lines, indicating that GDF15 can reverse the effects of ROS in the suppression of cancer stemness. Taken together, these studies suggest that GDF15 inhibits cellular ROS generation, which also promotes cancer stemness.

### GDF15 regulates cellular functions via a SMAD-associated signaling pathway

**Figure 5:**
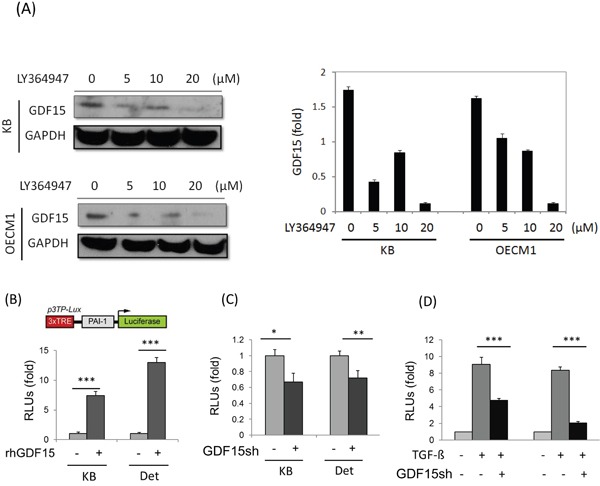
GDF15 regulates cellular functions through similar downstream pathway of TGF-β **A.** GDF15 expression was reduced after treatment of TGF-β inhibitor. KB and OECM1 cells were treated with serial doses of LY364947 (5 to 20 μM) for 24 h. Cellular protein was extracted and subjected to western blot analysis to assess GDF15 protein expression. **B.** Administration of rhGDF15 increased TGF-β downstream molecule PAI-1 luciferase reporter activity. KB or Detroit cells were transfected with luciferase reporter plasmid carrying PAI-1 gene, with or without addition of rhGDF15 (20 ng/ml). After 48 h, cells were harvested for measurements of luciferase activity (n=3). **C.** Silencing rhGDF15 suppressed PAI-1 luciferase reporter activity. KB or Detroit cells were transfected with luciferase reporter plasmid carrying PAI-1 gene, with or without co-transfection of GDF15sh plasmid. After 48 h, cells were harvested for measurements of luciferase activity (n=3). **D.** PAI-1 expression induced by TGF-β was suppressed following GDF15 silencing. KB or Detroit cells were transfected with luciferase reporter plasmid carrying PAI-1 gene, with or without addition of rhGDF15 (20 ng/ml), or with or without co-transfection of GDF15sh plasmid. After 48 h, cells were harvested for measurements of luciferase activity (n=3). (*: *p* < 0.05, **: *p* < 0.01, ***: *p* < 0.001, *t*-test).

Because GDF15 is a member of the TGF-β superfamily, we examined whether GDF15 regulates cellular functions through similar downstream pathway of TGF-β. The HNC cells were first treated with a TGF-β inhibitor, LY364947 [37], and the GDF15 level was examined. As shown in Figure 5A, the GDF15 expression was reduced in a dose-dependent manner in both HNC cell lines. To confirm the downstream molecular effects of GDF15, a PAI-1 luciferase reporter assay was used, which assessed the well-known TGF-β-inducing molecule, plasminogen activator inhibitor-1 (PAI-1) [38]. Administration of rhGDF15 significantly increased PAI-1 luciferase activity by 8- to 13-fold in two HNC cell lines (Figure 5B), whereas GDF15 silencing suppressed this activation to 64% to 69% of the controls (Figure 5C). Furthermore, the PAI-1 expression induced by TGF-β was strongly suppressed following GDF15 silencing, by 45% to 71% in two HNC cell lines (Figure 5D). These results suggested that GDF15 may have a similar molecular pathway as TGF-β, and cross-talk with the TGF-β regulatory network may occur in HNC cells.

**Figure 6:**
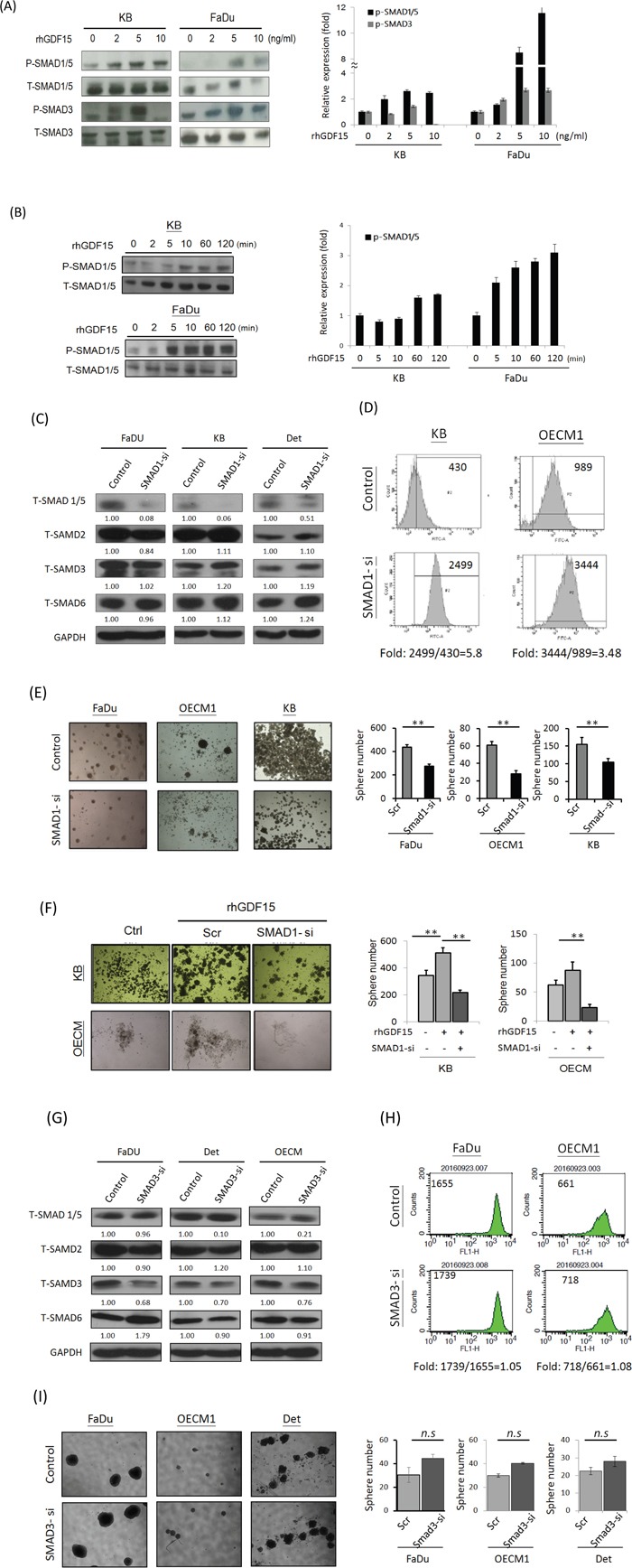
GDF15 regulates cellular functions via a SMAD-associated signaling pathway **A, B.** Administration of rhGDF15 increased the phosphorylated forms of SMAD family proteins in HNC cells. KB or FaDu cells were treated with serial doses of rhGDF15 (0-10 ng/ml) for 15 min (A) or 5 ng/ml rhGDF15 for various times (0-120 min) (n=3). (B) The cellular proteins were extracted and subjected to western blot analysis for SMAD family protein expressions (n=3). **C.** Effects of SMAD1-siRNA on the expressions of SMAD family proteins. After transfection of SMAD1-specific siRNA or the scramble oligonucleotides in HNC cells for 48h, cellular proteins were extracted for western blot analysis. GAPDH protein was used as an internal control (n=3). **D.** Silencing SMAD1 increased ROS level in HNC cells. KB or OECM1 cells were transfected SMAD1 specific siRNA or the scramble oligonucleotides for 48 h. After treating cells with 10 μM H_2_O_2_ for 20 min, the Intracellular ROS levels were determined by DCF dye staining and analyzed with flow cytometry. **E.** Silencing SMAD1 suppressed spheroid cell formation in HNC cells. Fadu, OECM1 or KB cells were transfected SMAD1 specific siRNA or the scramble oligonucleotides for 48 h. These cells were then incubated in the spheroid cell culture condition and assessed after 14 days (n=3). **F.** The spheroid cell formation promoted by GDF15 was inhibited in SMAD knockdown HNC cells. KB or OECM1 cells were transfected SMAD1 specific siRNA or the scramble oligonucleotides for 48 h, with the addition of rhGDF15 (20 ng/ml). These cells were then incubated in the spheroid cell culture condition and assessed after 14 days (n=3). **G.** Effects of SMAD3-siRNA on the expressions of SMAD family proteins. After transfection of SMAD3-specific siRNA or the scramble oligonucleotides in HNC cells for 48h, cellular proteins were extracted for western blot analysis. GAPDH protein was used as an internal control (n=3). **H.** Silencing SMAD3 had no effect on ROS level in HNC cells. Fadu or OECM1 cells were transfected SMAD3-specific siRNA or the scramble oligonucleotides for 48 h. After treating cells with 10 μM H_2_O_2_ for 20 min, the Intracellular ROS levels were determined by DCF dye staining and analyzed with flow cytometry. **I.** Silencing SMAD3 had no significant effect on spheroid cell formation in HNC cells. Fadu, OECM1 or Detroit cells were transfected SMAD3 specific siRNA or the scramble oligonucleotides for 48 h, with the addition of rhGDF15 (20 ng/ml). After 14 days of incubation in the spheroid cell culture condition, cells were assessed for spheroid formation (n=3). (*: *p* < 0.05, **: *p* < 0.01, ***: *p* < 0.001, *n.s.*: non-significance, *t*-test).

The SMAD family plays a key role as downstream molecules of TGF-β, which regulates the pluripotent stemness phenotype [39, 40]. We therefore examined whether GDF15 functions in HNC cells via a SMAD-associated signaling pathway. Administration of rhGDF15 significantly increased the phosphorylated forms of SMAD family proteins, with pSMAD1/5 the most prominent (Figure 6A). This activation of pSMAD1/5 by GDF15 was shown to be time-dependent in two HNC cell lines (Figure 6B). These results indicated that GDF15 functions in cells through phosphorylation of SMAD1/5 molecules to activate the downstream signaling pathway.

We further examined whether GDF15 contributed to ROS suppression and cancer stemness through the SMAD regulatory pathway. As shown, specific knockdown SMAD1 (Figure 6C) substantially increased ROS levels (Figure 6D) and suppressed cancer stemness (Figure 6E). Additionally, the cancer stemness phenotype promoted by GDF15 was significantly inhibited in SMAD1 knockdown cells, as shown in two HNC cell lines (Figure 6F). However, knockdown SMAD3 (Figure 6G) had minimal effect on either ROS level (Figure 6H) or cancer stemness formation (Figure 6I). These results suggested that GDF15 regulations cell function via a via a SMAD1-associated signaling pathway.

### GDF15 promotes radioresistant tumors in mice, along with SMAD activation and stemness conversion

**Figure 7:**
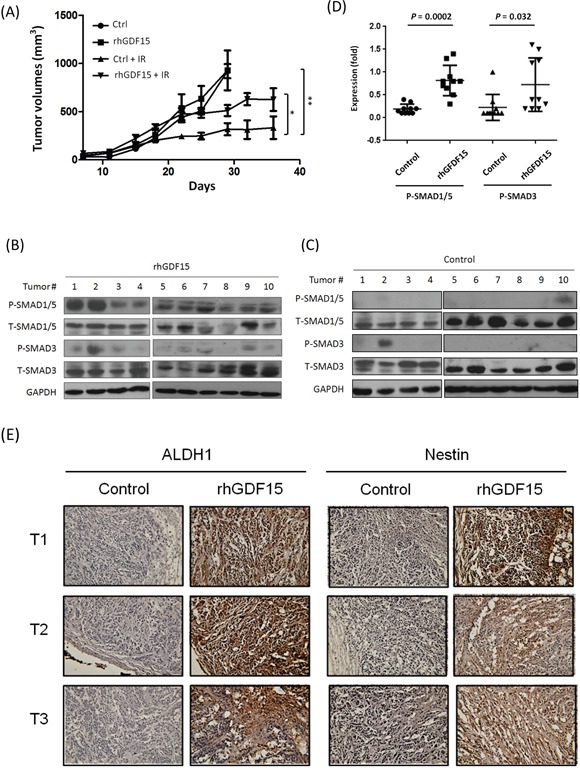
GDF15 promotes radioresistant tumors in mice, along with SMAD activation and stemness conversion A total of 4×10^6^ KB cells, with or without pre-treatment with the rhGDF15 protein (20 ng/ml for 5 days), were subcutaneously injected into BALB/c mice (10 mice each group) in the upper portion of the hind limb. At day 14, each group was randomly divided into two groups (5 mice per group), with or without receiving 2 Gy of irradiation, followed by repeated irradiation of the same dose twice a week for a total of 8 Gy. **A.** Tumor volume was measured twice a week and calculated as (length x width x height) for 36 days. **B-D.** The tumors in the group of irradiation, either with or without pre-treatment of rhGDF15, were dissected. The protein expression levels of SMAD family molecules in the rhGDF15 treatment tumor group (B) or the control groups (C) were determined by using western blot analysis, and quantified the relative expression levels after normalized with GAPDH (D). **E.** The expression levels of ALDH1 and Nestin in tumor tissues were determined by using IHC analysis. Three tumor sections of IHC staining were shown for examples (*: *p* < 0.05, **: *p* < 0.01, ***: *p* < 0.001, *t*-test).

To examine the effect of GDF15 on radioresistance in vivo, we established xenografted tumors in BALB/c nude mice. The HNC cells were first treated with or without rhGDF15 before inoculation into mice. To establish tumors in mice, two groups of mice were inoculated with rhGDF-treated cells, followed with or without radiation treatment. Another two groups were inoculated with control HNC cells, followed with or without radiation treatment. These mice were continuously monitored for up to 36 days. The tumor growth results are shown in Figure 7A. In the absence of radiation, there was no statistical difference in tumor growth rates between the with- and without-rhGDF15 treatment groups. After irradiation, although inhibition of tumor growth was found in both groups, tumors in the rhGDF15-treatment group grew much faster than those in the control group and were approximately 2-fold larger in size after 3 weeks (*P* = 0.016 at day 36). The results demonstrated that GDF15 confers resistance to the radiation treatment.

To determine whether the differential tumor growth associated with the rhGDF15 treatment was related to SMAD1 activation and stemness conversion, the xenografted tumors were dissected, and protein expression was examined using western blot and immunohistochemistry assays. The results of the two sets of xenografted tumors receiving irradiation were shown in Figure 7B-7D. Although there was variation between individual tumors, the pSMAD1/5 and pSMAD3 levels were generally higher in the rhGDF15-treated xenografts (Figure 7B) compared to the controls (Figure 7C), with average elevations of 2.0-fold (*P* = 0.0002) for pSMAD1/5 and 1.6-fold (*P* = 0.032) for the pSMAD3 proteins (Figure 7D). The results of immunohistochemistry staining for the cancer stemness marker proteins ALDH1 and Nestin in the dissected xenograft tumors are shown in Figure 7E. In all tumors examined, the rhGDF15-treated tumors exhibited a strong staining of these two proteins in the entire tumor mass compared to the controls. These results suggested that GDF15 conferred radioresistance in vivo through SMAD activation and stemness conversion.

## DISCUSSION

Radiotherapy is an integral part of the treatment of HNC. Understanding the molecular mechanisms associated with radioresistance will help to improve the efficacy of radiotherapy. Previously, GDF15 was reported to be associated with chemo-radioresistance. The concordant findings showed an increase in GDF15 expression in irradiated oral cancer cells [41], an increase in plasma GDF15 levels in chemotherapeutic-resistant testicular cancer patients [42], and increased sensitivity to chemo-drug treatment after GDF15 knockdown in a mouse model of ovarian cancer [43]. However, an adverse function of GDF15 has also been reported, as GDF15 leads to cellular senescence in response to irradiation in endothelial cells [27]. These conflicting results may be due to differential tissue specificity, the distinct tumor status or the microenvironment that has not yet been defined. Consistent with other reports, we previously observed that GDF15 is up-regulated in HNC cell lines with high radioresistant properties [6, 7]. In the present study, we further showed that GDF15 actively contributed to radioresistance (Figure 1A-1C) in HNC but had no function in cell growth (Figure 1D), as shown in both cellular (Figure 1) and animal model studies (Figure 7A). These results demonstrate the significance of GDF15 levels on the efficacy of radiotherapy in HNC.

A model of cancer stem cells has been recently proposed to explain tumor heterogeneity. These cells have been hypothesized to possess a strong malignant potential, with high mobility, the capacity for self-renewal, and stress tolerance, which results in resistance to chemo-radiotherapy [31–33]. These stem types of cells are often characterized by specific surface proteins, such as CD44 and ALDH1, in head and neck tissues [31–33]. We therefore investigated whether GDF15 has a role in the regulation of cancer stemness in HNC. For the stemness phenotype, GDF15 has been previously reported to participate in the cellular differentiation of osteoblasts [44, 45] and to enhance tumor initiation and malignancy in multiple myeloma cells [46]. Consistent with these studies, we found that GDF15 promotes cancer stemness, as shown by the increase in spheroid cell formation (Figure 3I-3J) and invasion ability (Figure 3K-3L). Furthermore, these functions were accompanied by increases in the CD44+ and ALDH+ cellular populations (Figure 3A-3H). Therefore, GDF15 contributes to the malignancy of HNC by facilitating the conversion to the CD44+/ALDH+ stem cell-like phenotype.

Cellular ROS are the most crucial factor leading to cancer cell apoptosis after irradiation treatment, whereas low ROS is the major cause of radioresistance [29, 47]. Recently, several studies also showed a functional connection between intracellular ROS levels and the stemness status of cells. All stem cells, whether pluripotent or multipotent, appear to have lower cellular ROS levels to maintain their genomic integrity [48]. Similarly, cancer stem cells often display very low levels of ROS, mainly due to the increased activity of the antioxidant machinery in tumors [48, 49]. In this study, we confirmed that lower ROS levels contribute to radioresistance (Figure 4A) and cancer stemness (Figure 4B) in HNC. We further found that GDF15 reduced cellular ROS production, as shown by the positive (Figure 2C) and negative (Figure 2D) regulatory models. We also demonstrated that GDF15 was able to rescue the effects of ROS-induced cellular alteration (Figure 4C). Consistent with our findings, GDF15 has been reported to play a cytoprotective role against high glucose-induced cell apoptosis by inhibiting ROS production [50]. Thus, GDF15 contributes to radioresistance and cancer stemness in HNC through mechanisms involving in the inhibition of cellular ROS production.

Cysteine knots of the GDF15 structure represent a highly conserved sequence of the TGF-β super-family [8–9]. In the present study, we found that there may be cross-talk between GDF15 and the TGF-β signaling pathway in HNC cells, as shown by the regulation of a TGF-β inhibitor (Figure 5A), and the effects on TGF-β downstream molecules (Figure 5B-5C). In support of this hypothesis, the TGF-β pathway has previously been reported to possess similar functions to those we found in GDF15, as promoting the radioresistant phenotype [51], reducing intracellular ROS level [52], and contributing to cancer stemness [53]. Therefore, GDF15 and TGF-β may share a common signaling pathway or participate in a mutual regulatory network in cells.

The cellular functions of GDF15 are effected through various downstream molecular mechanisms. For example, GDF15 stimulated cell proliferation via PI3K/Akt and Erk signaling pathways in endothelial cells [54]. These Akt and Erk pathways have also been reported to induce cell growth by GDF15 in esophageal and ovarian cancers [22, 55]. GDF15 promoted metastasis through the FAK-RhoA signaling pathway in prostate cancer [56], whereas this metastatic function was achieved through Smad2/3 signaling in colorectal cancer [57]. In the present study, we found that GDF15 facilitated cancer stemness and radioresistance via activation of Smad1/5, as demonstrated in both cellular (Figure 6C-6F) and animal studies (Figure 7B-7D). Consistently, this Smad-associated pathway has been shown to be induced by GDF15 with anti-apoptotic effects in cardiomyocytes [58, 59]. Thus, GDF15 may have multiple functions in cells through distinctive signaling pathways depending on the specific tissue types.

Aggressive tumors selectively enhance particular characteristics in progressing to a therapy-refractive state. To elucidate whether GDF15 may contribute to the resistance of radiotherapy in vivo, we performed xenograft tumorigenic studies. Although tumor growth showed no difference in the absence of radiation, we found that tumors with higher GDF15 exhibited significant resistance to radiation treatment (Figure 7A). These results were accompanied with the augmentation of Smad protein phosphorylations (Figure 7B-7D) and elevated expression of the cancer stemness markers ALDH1 and Nestin (Figure 7E). These results were consistent with cellular findings that GDF15 rendered a more prominent effect on SMAD1/5 phosphorylation. The induction of pSMAD3 in vivo may be required by the collaboration of cellular mechanisms with tumor niche.

**Figure 8:**
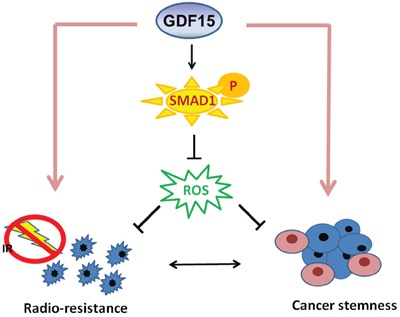
Diagram of the mechanism by which GDF15 contributes to radioresistance and cancer stemness through regulating ROS levels via a SMAD-associated pathway

Taken together, our results showed that GDF15 contributed to HNC by facilitating cancer stemness. This phenotype may be achieved by regulation of intracellular ROS levels via a SMAD1-associated signaling pathway (Figure 8). GDF15 may serve as a predictive marker of radioresistance and a molecular target for the development of a therapeutic modality for eradiating refractory HNC.

## MATERIALS AND METHODS

### Cell culture and spheroid cell formation

HNC cell lines, including KB, OECM1, Detroit and Fadu, were used. Cells were maintained as previously described [7, 60]. All cells were grown at 37°C in a humidified incubator containing 5% CO_2_. The spheroid cell formation assay was performed as previously described [33, 34]. Briefly, cells were cultured in a suspension (3,000-5,000 cells/ml) with F12/DMEM medium containing 1% Matrigel, N2 supplement, 10 ng/ml EGF, and 10 ng/ml bFGF. After 2 weeks, cellular spheres were visualized, and 5 independent microscopic fields were examined.

### Silencing or modulation of GDF15 and SMAD molecules

The GDF15 shRNA plasmid was constructed as previously described [61]. In brief, the 22 nucleotide sense and antisense hairpin oligonucleotides were designed to be complementary to the GDF15 mRNA sequence 5′-GGA- UAC- UCA- CGC- CAG- AAG- UGCG-3′. The GDF15-shRNA was then cloned into the pTopo-U6 vector plasmid. The SMAD1-specific siRNA (#6223S) was purchased from Cell Signaling Technology (Danver, MA, USA). The plasmid transfection was performed using a Nucleofector™ Kit (Lonza, Basel, Switzerland) according to the manufacturer's instructions. For administration of GDF15, the recombinant human GDF15 protein (rhGDF15) (#957-GD, R&D Systems, Minneapolis, MN) was added to the cell culture medium at 20 ng/ml for 2 to 5 days.

### Analysis of CD44^+^ and ALDH1^+^ fractions by flow cytometry

Fluorescence-activated cell sorting (FACS) was used to isolate specific fractions of the cell population as previously described [33]. Briefly, the HNC cells in suspension were incubated with FITC-conjugated CD44 antibodies or the DEAB- or FITC-conjugated ALDH1 antibodies (Aldefluor kit, Stem Cell Technology, Vancouver, BC, Canada) for 60 min. After washing, the antibody-labeled cells were analyzed by flow cytometry (FACSCalibur, BD Biosciences, Franklin Lakes, NJ). The ALDH+ population was normalized to the DEAB-labeled fraction. Cells were stained with isotype-matched antibodies, and cells lacking primary antibody labeling were used as negative controls.

### Determination of radiosensitivity and cell invasion ability

Radiosensitivity was determined by clonogenic survival assays as previously described [62]. Briefly, a total of 800 cells was seeded into a 6-well plate and irradiated with serial doses of radiation (0 to 6 Gy). The cells were cultured for 12-14 days, followed by the calculation of the number of surviving colonies. The survival fraction was calculated as the number of colonies divided by the number of cells seeded times plating efficiency. Cell invasion assays were performed using BioCoat Matrigel and Transwell invasion chambers (Millipore Corp, Billerica, MA) as previously described [63]. The invaded cells that passed through the Matrigel-coated membranes to the reverse side were stained and photographed.

### RNA extraction and RT- PCR analysis

The RNA extraction, cDNA synthesis and PCR reaction were performed as previously described [64]. These PCR products were analyzed by 1.5% agarose gel electrophoresis. The density of each band was quantified after normalization to the actin control band using the Gel Image System (Scion Corporation, MD). All the experiments were performed duplicate for three times. The error bars shown in the relevant figures indicated the standard deviation of the quantification results in all experiments. The primers used in this study for GDF15, CD44, and actin are listed in Supplemental Table S1.

### Protein extraction and western blot analysis

The protein extraction and western blot analysis were performed as previously described [65]. Briefly, cellular proteins were separated by SDS-polyacrylamide gel electrophoresis and transferred to a nitrocellulose membrane. The membrane was hybridized with primary antibodies and then incubated with horseradish peroxidase-conjugated secondary antibodies. The membranes were developed using an ECL developing solution (Amersham Pharmacia Biotech, Piscataway, NJ, USA) followed by autoradiography. All the experiments were performed three times independently and that typical results were shown. Using the Gel Image System (Scion Corporation, MD), the density of each band was quantified after normalization to the GAPDH control band. The error bars shown in the relevant figures indicated the standard deviation of the quantification results in all experiments. The primary antibodies used in this study, including antibodies against GDF15, CD44, ALDH1, Nestin, ph-SMAD1/5, SMAD1/5, ph-SMAD3, SMAD3, and GAPDH, are listed in Supplemental Table S2.

### Measurement of reactive oxygen species

Intracellular reactive oxygen species (ROS) were measured by the H_2_DCF-DA oxidation method (Invitrogen, Carlsbad, CA, USA) as previously described [34]. Briefly, cells were grown on plates or coverslips in HEPES buffer supplemented with H_2_DCF-DA reagent. The H_2_DCF-DA is a cell-permeable probe that is oxidized by intracellular ROS to generate fluorescent DCF. The green fluorescence of DCF was monitored by confocal laser microscopy (Zeiss Microscopy GmbH, Jena, Germany) or flow cytometric analysis (FACSCalibur, BD Biosciences, Franklin Lakes, NJ, USA). The experiments were performed three times independently. All similar results were obtained and a typical data was shown.

### Detection of mitochondrial membrane potential

The mitochondrial membrane potential was determined using a MitoCapture™ Apoptosis Detection Kit (Biovision, Milpitas, CA, USA) according to the manufacturer's suggested protocol. The kit utilizes a cationic dye that detects changes in the mitochondrial transmembrane potential in healthy (red fluorescence) and in apoptotic (green fluorescence) cells. The intensity of cellular fluorescence was analyzed by fluorescence microscopy or by flow cytometry (FACSCalibur, BD Biosciences). The experiments were performed three times independently. All similar results were obtained and a typical data was shown.

### Luciferase reporter assay for determination of TGF-β activity

The TGF-β activity was measured based on its ability to induce plasminogen activator inhibitor-1 (PAI-1) expression, which was measured with a PAI-1 luciferase reporter assay (Addgene, Cambridge, MA, USA). The luciferase reporter plasmid containing PAI-1 binding sites was transfected into cells. After modulation of TGF-β or GDF15 expression by transfection of the specific shRNA or the addition of the recombinant proteins (TGF-β, #240-B or GDF15, #957-GD, R&D Systems, Minneapolis, MN), the luciferase activity in each experiment was determined as previously described [66]. All the experiments were performed triplicate for three times and the similar results were obtained. The error bars shown in the relevant figures indicated the standard deviation of a triplicate experiment.

### Xenografts and in vivo tumorigenicity

All animal studies were approved by the Chang Gung University Animal Care Committee. A total of 20 immunodeficient mice (BALB/cAnN.Cg-*Foxnl^nu^*/CrlNarl, 4 to 5 weeks old) were used, and the xenograft experiments were performed as previously described [7, 63]. Briefly, a total of 4 × 10^6^ KB cells, with or without pre-treatment with the rhGDF15 protein (20 ng/ml for 5days), were subcutaneously injected into BALB/c mice (10 mice each group) in the upper portion of the hind limb. At day 14, each group was randomly divided into two groups (5 mice per group), with or without receiving 2 Gy of irradiation, followed by repeated irradiation of the same dose twice a week for a total of 8 Gy. Tumor volume was measured twice a week and calculated as (length x width x height). On day 36, the mice were euthanized, and the tumors were removed for IHC and western blot analyses.

### Immunohistochemical analysis

Immunohistochemistry (IHC) was performed as previously described [67]. Briefly, tumors from xenografts were fixed in 10% neutralized buffered formaldehyde, embedded in paraffin, and then stained with anti-ALDH1 or anti-Nestin antibodies. The sources of these primary antibodies are listed in Supplemental Table S2. IHC analysis and color development were performed using the AEC substrate chromogen system (LSAB2 System; Dako, Agilent Technologies, Santa Clara, CA) following the manufacturer's instruction and a light microscope.

### Statistical analysis

The *t*-test was used to analyze the statistical significance between experiments. Significance was considered when *p* value was lower than 0.05.

## SUPPLEMENTARY TABLES



## References

[1] Saman DM (2012). A review of the epidemiology of oral and pharyngeal carcinoma: update. Head Neck Oncol.

[2] Chen YJ, Chang JT, Liao CT, Wang HM, Yen TC, Chiu CC, Lu YC, Li HF, Cheng AJ (2008). Head and neck cancer in the betel quid chewing area: recent advances in molecular carcinogenesis. Cancer Sci.

[3] Stucken E, Weissman J, Spiegel JH (2010). Oral cavity risk factors: experts' opinions and literature support. J Otolaryngol Head Neck Surg.

[4] Higgins GS1, O'Cathail SM2, Muschel RJ3, McKenna WG (2015). Drug radiotherapy combinations: Review of previous failures and reasons for future optimism. Cancer Treat Rev.

[5] Klein J, Livergant J, Ringash J (2014). Health related quality of life in head and neck cancer treated with radiation therapy with or without chemotherapy: a systematic review. Oral Oncol.

[6] Chang JT, Chan SH, Lin CY, Lin TY, Wang HM, Liao CT, Wang TH, Lee LY, Cheng AJ (2007). Differentially expressed genes in radioresistant nasopharyngeal cancer cells: gp96 and GDF15. Mol Cancer Ther.

[7] Lin TY, Chang JT, Wang HM, Chan SH, Chiu CC, Lin CY, Fan KH, Liao CT, Chen IH, Liu TZ, Li HF, Cheng AJ (2010). Proteomics of the radioresistant phenotype in head-and-neck cancer: gp96 as a novel prediction marker and sensitizing target for radiotherapy. Int. J. Radiation Oncology Biol.

[8] Unsicker K, Spittau B, Krieglstein K (2013). The multiple facets of the TGF-β family cytokine growth/differentiation factor-15/macrophage inhibitory cytokine-1. Cytokine Growth Factor Rev.

[9] Corre J, Hebraud B, Bourin P (2013). Concise review: growth differentiation factor 15 in pathology: a clinical role?. Stem Cells Transl Med.

[10] Adela R, Banerjee SK (2015). GDF-15 as a target and biomarker for diabetes and cardiovascular diseases: a translational prospective. J Diabetes Res.

[11] Li C, Wang X, Casal I, Wang J, Li P, Zhang W, Xu E, Lai M, Zhang H (2016). Growth differentiation factor 15 is a promising diagnostic and prognostic biomarker in colorectal cancer. J Cell Mol Med.

[12] Mehta RS, Chong DQ, Song M, Meyerhardt JA, Ng K, Nishihara R, Qian Z, Morikawa T, Wu K, Giovannucci EL, Fuchs CS, Ogino S, Chan AT (2015). Association between plasma levels of marcrophage inhibitory cytokine-1 before diagnosis of colorectal cancer and mortality. Gastroenterology.

[13] Kaur S, Chakraborty S, Baine MJ, Mallya K, Smith LM, Sasson A, Brand R, Guha S, Jain M, Wittel U, Singh SK, Batra SK (2013). Potentials of plasma NGAL and MIC-1 as biomarker(s) in the diagnosis of lethal pancreatic cancer. PLoS One.

[14] Mohamed AA, Soliman H, Ismail M, Ziada D, Farid TM, Aref AM, Al Daly ME, Abd Elmageed ZY (2015). Evaluation of circulating ADH and MIC-1 as diagnostic markers in Egyptian patients with pancreatic cancer. Pancreatology.

[15] Wang X, Li Y, Tian H, Qi J, Li M, Fu C, Wu F, Wang Y, Cheng D, Zhao W, Zhang C, Wang T, Rao J, Zhang W (2014). Marcophage inhibitory cytokine (MIC-1/GDF15) as a novel diagnostic serum biomarker in pancreatic ductal adenocarcinoma. BMC Cancer.

[16] Fisher OM, Levert-Mignon AJ, Lord SJ, Lee-Ng KK, Botelho NK, Falkenback D, Thomas ML, Bobryshev YV, Whiteman DC, Brown DA, Breit SN, Lord RV (2015). MIC-1/GDF15 in Barrett's oesophagus and oesophageal adenocarcinoma. Br J Cancer.

[17] Liu X, Chi X, Gong Q, Gao L, Niu Y, Chi X, Cheng M, Si Y, Wang M, Zhong J, Niu J, Yang W (2015). Association of serum level of growth differentiation factor 15 with liver cirrhosis and hepatocellular carcinoma. PLoS One.

[18] Wang XB, Jiang XR, Yu XY, Wang L, He S, Feng FY, Guo LP, Jiang W, Lu SH (2014). Macrophage inhibitory factor 1 acts as a potential biomarker in patients with esophageal squamous cell carcinoma and is a target for antibody-based therapy. Cancer Sci.

[19] Yang CZ, Ma J, Luo QQ, Neskey DM, Zhu DW, Liu Y, Myers JN, Zhang CP, Zhang ZY, Zhong LP (2014). Elevated level of serum growth differentiation factor 15 is associated with oral leukoplakia and oral squamous cell carcinoma. J Oral Pathol Med.

[20] Trovik J, Salvesen HB, Cuppens T, Amant F, Staff AC (2014). Growth differentiation factor-15 as biomarker in uterine sarcomas. Int J Gynecol Cancer.

[21] Blanco-Calvo M, Tarrío N, Reboredo M, Haz-Conde M, García J, Quindós M, Figueroa A, Antón-Aparicio L, Calvo L, Valladares-Ayerbes M (2014). Circulating levels of GDF15, MMP7 and miR-200c as a poor prognostic signature in gastric cancer. Future Oncol.

[22] Urakawa N, Utsunomiya S, Nishio M, Shigeoka M, Takase N, Arai N, Kakeji Y, Koma Y, Yokozaki H (2015). GDF15 derived from both tumor-associated marcophages and esophageal squamous cell carcinomas contributes to tumor progression via Akt and Erk pathway. Lab Invest.

[23] Zhang Y, Hua W, Niu LC, Li SM, Wang YM, Shang L, Zhang C, Li WN, Wang R, Chen BL, Xin XY, Zhang YQ, Wang J (2016). Elevated growth differentiation factor 15 expression predicts poor prognosis in epithelial ovarian cancer patients. Tumour Biol.

[24] Staff AC, Trovik J, Eriksson AG, Wik E, Wollert KC, Kempf T, Salvesen HB (2011). Elevated plasma growth differentiation factor-15 correlates with lymph node metastases and poor survival in endometrial cancer. Clin Cancer Res.

[25] Qian Y, Jung YS, Chen X (2012). Differentiated embryo-chondrocyte expressed gene 1 regulates p53-dependent cell survival versus cell death through macrophage inhibitory cytokine-1. Proc Natl Acad Sci USA.

[26] Li PX, Wong J, Ayed A, Ngo D, Brade AM, Arrowsmith C, Austin RC, Klamut HJ (2000). Placental transforming growth factor-b is a downstream mediator of the growth arrest and apoptotic response of tumor cells to DNA damage and p53 overexpression. J Biol Chem.

[27] Park H, Kim CH, Jeong JH, Park M, Kim KS (2016). GDF15 contributes to radiation-induced sensescence through the ROS-mediated p16 pathway in human endothelial cells. Oncotarget.

[28] Tsui KH, Hsu SY, Chung LC, Lin YH, Feng TH, Lee TY, Chang PL, Juang HH (2015). Growth differentiation factor-15: a p53- and demethylation- upregulating gene represses cell proliferation, invasion, and tumorigenesis in bladder carcinoma cells. Sci Rep.

[29] Diehn M, Cho RW, Lobo NA, Kalisky T, Dorie MJ, Kulp AN, Qian D, Lam JS, Ailles LE, Wong M, Joshua B, Kaplan MJ, Wapnir I (2009). Association of reactive oxygen species levels and radioresistance in cancer stem cells. Nature.

[30] Visagie MH, Joubert AM (2011). In vitro effects of 2-methoxyestradiol-bis-sulphamate on reactive oxygen species and possible apoptosis induction in a breast adenocarcinoma cell line. Cancer Cell Int.

[31] Buto R, Dubrovska A, Baumann M (2013). Clinical perspectives of cancer stem cell research in radiation oncology. Radiother Oncol.

[32] Yang C, Jin K, Tong Y, Cho WC (2015). Therapeutic potential of cancer stem cells. Med Oncol.

[33] Chiu CC, Lee LY, Li YC, Chen YJ, Lu YC, Li YL, Wang HM, Chang JT, Cheng AJ (2013). Grp78 as a therapeutic target for refractory head-neck cancer with CD24-CD44+ stemness phenotype. Cancer Gene Ther.

[34] Li YC, Chang JT, Chiu C, Lu YC, Li YL, Chiang CH, You GR, Lee LY, Cheng AJ (2016). Areca nut contributes to oral malignancy through facilitating the conversion of cancer stem cells. Mol Carcinog.

[35] Isah MB, Ibrahim MA (2014). The role of antioxidants treatment on the pathogenesis of malarial infections: a review. Parasitol Res.

[36] Li J, Meng Z, Zhang G, Xing Y, Feng L, Fan S, Fan F, Buren B, Liu Q (2015). N-acetylcysteine relieves oxidative stress and protects hippocampus of rat from radiation-induced apoptosis by inhibiting caspase-3. Biomed Phamacother.

[37] Kano MR, Komuta Y, Iwata C, Oka M, Shirai YT, Morishita Y, Ouchi Y, Kataoka K, Miyazono K (2009). Comparison of the effects of the kinase inhibitors imathinib, sorafenib, and transforming growth factor-beta receptor inhibitor on extravasation of nanoparticles from noevasculature. Cancer Sci.

[38] Abe M, Harpel JG, Metz CN, Nunes I, Loskutoff DJ, Rifkin DB (1994). An assay for transforming growth factor-β using cells transfected with a plasminogen activator inhibitor-1 promoter- luciferase construct. Anal Biochem.

[39] Heldin CH, Landstrom M, Moustakas A (2009). Mechanism of TGF-β signaling to growth arrest, apopotsis, and epithelial-mesenchymal transition. Curr Opin Cell Biol.

[40] Gaarenstroom T, Hill CS (2014). TGF-b signaling to chromatin: how Smads regulate transcription during self-renewal and differentiation. Semin Cell Dev Biol.

[41] Schiegnitz E, Kammerer PW, Rode K, Schorn T, Brieger J, Al-Nawas B (2016). Growth differentiation factor 15 as a radiation-induced marker in oral carcinoma increasing radiation resistance. J Oral Pasthol Med.

[42] Altena R, Fehrmann RS, Boer H, de Vries EG, Meijer C, Gietema JA (2015). Growth differentiation factor 15 (GDF-15) plasma levels increase during bleomycin- and cisplatin-based treatment of testicular cancer patients and relate to endothelial damage. PLoS One.

[43] Meier JC, Haendler B, Seidel H, Groth P, Adams R, Ziegelbauer K, Kreft B, Beckmann G, Sommer A, Kopitz C (2015). Knockdown of platinum- induced growth differentiation factor 15 abrogates p27- mediated tumor growth delay in the chemoresistant ovarian cancer model A2780cis. Cancer Med.

[44] Westhrin M, Moen SH, Holien T, Mylin AK, Heickendorff L, Olsen OE, Sundan A, Turesson I, Gimsing P, Waage A, Standal T (2015). Growth differentiation factor15 (GDF15) promotes osteoclast differentiation and inhibits osteoblast differentiation and high serum GDF15 levels are associated with multiple myeloma bone disease. Haematologica.

[45] Uchiyama T, Kawabata H, Miura Y, Yoshioka S, Iwasa M, Yao H, Sakamoto S, Fujimoto M, Haga H, Kadowaki N, Maekawa T, Takaori-Kondo A (2015). The role of growth differentiation factor 15 in the pathogenesis of primary myelofibrosis. Cancer Med.

[46] Tanno T, Lim Y, Wang Q, Chesi M, Bergsagel PL, Matthews G, Johnstone RW, Ghosh N, Borrello I, Huff CA, Matsui W (2014). Growth differentiating factor 15 enhances the tumor-initiating and self-renewal potential of multiple myeloma cells. Blood.

[47] Ozben T (2007). Oxidative stress and apoptosis: Impact on cancer therapy. J Pharm Sci.

[48] Chaudhari P, Ye Z, Jang YY (2014). Roles of reactive oxygen species in the fate of stem cells. Antioxid Redox Signal.

[49] Bigarella C, Liang R, Ghaffari S (2014). Stem cells and the impact of ROS signaling. Development.

[50] Li J, Yang L, Qin W, Zhang G, Yuan J, Wang F (2013). Adaptive induction of growth differentiation factor 15 attenuates endothelial cell apoptosis in response to high glues stimulus. PLoS One.

[51] Zhang M, Kleber S, Röhrich M, Timke C, Han N, Tuettenberg J, Martin-Villalba A, Debus J, Peschke P, Wirkner U, Lahn M, Huber PE (2011). Blockade of TGF-β signaling by the TGFβR-I kinase inhibitor LY2109761 enhances radiation response and prolongs survival in glioblastoma. Cancer Res.

[52] Kajino-Sakamoto R, Omori E, Nighot PK, Blikslager AT, Matsumoto K, Ninomlya-Tsuji J (2010). TGF-beta-activated kinase 1 signaling maintains intestinal integrity by preventing accumulation of reactive oxygen species in the intestinal epithelium. J Immunol.

[53] Wang M, Hada M, Huff J, Pluth JM, Anderson J, O'Neill P, Cucinotta FA (2012). Heavy ions can enhance TGF-β mediated epithelial to mesenchymal transition. J Radiat Res.

[54] Jin YJ, Lee JH, Kim YM, Oh GT, Lee H (2012). Macrophage inhibitory cytokine-1 stimulates proliferation of human umbilical vein endothelial cells by up-regulating cyclins D1 and E through the PI3K/Akt-, ERK-, and JNK-dependent AP-1 and E2F activation signaling pathways. Cell Signal.

[55] Griner SE, Joshi JP, Nahta R (2013). Growth differentiation factor 15 stimulates rapamycin- sensitive ovarian cancer cell growth and invasion. Biochem Pharmacol.

[56] Senapati S, Rachagani S, Chaudhary K, Johansson SL, Singh RK, Batra SK (2010). Overexpression of macrophage inhibitory cytokine-1 induces metastasis of human prostate cancer cells through the FAK-RhoA signaling pathway. Oncogene.

[57] Li C, Wang J, Kong J, Tang J, Wu Y, Xu E, Zhang H, Lai M (2016). GDF15 promotes EMT and metastasis in colorectal cancer. Oncotarget.

[58] Xu J, Kimball TR, Lorenz JN, Brown DA, Bauskin AR, Klevitsky R, Hewett TE, Breit SN, Molkentin JD (2006). GDF15/MIC-1 functions as a protective and antihypertrophic factor released from the myocardium in association with SMAD protein activation. Circ Res.

[59] Heger J, Schiegnitz E, von Waldthausen D, Anwar MM, Piper HM, Euler G (2010). Growth differentiation factor 15 acts anti-apoptotic and pro-hypertrophic in adult cardiomyocytes. J Cell Physiol.

[60] Chiu CC, Lin CY, Lee LY, Chen YJ, Lu YC, Wang HM, Liao CT, Chang JT, Cheng AJ (2011). Molecular chaperones as a common set of proteins that regulate the invasion phenotype of head and neck cancer. Clin Cancer Res.

[61] Chen YJ, Chang JT, Lee L, Wang HM, Liao CT, Chiu CC, Chen PJ, Cheng AJ (2007). DSG3 is overexpressed in head neck cancer and is a potential molecular target for inhibition of oncogenesis. Oncogene.

[62] Lu YC, Chen YJ, Wang HM, Tsai CY, Chen WH, Huang YC, Fan KH, Tsai CN, Huang SF, Kang CJ, Chang JT, Cheng AJ (2012). Oncogenic function and early detection potential of miRNA-10b in oral cancer as identified by microRNA profiling. Cancer Prev Res.

[63] Lu YC, Chang JT, Liao CT, Kang CJ, Huang SF, Chen IH, Huang CC, Huang YC, Chen WH, Tsai CY, Wang HM, Yen TC, You GR, Chiang CH, Cheng AJ (2014). Oncomir-196 promotes an invasive phenotype in oral cancer through the NME4-JNK-TIMP-MMP signaling pathway. Mol Cacer.

[64] Lin CY, Chen WH, Liao CT, Chen IH, Chiu CC, Wang HM, Yen TC, Lee LY, Chang JT, Cheng AJ (2010). Positive association of glucose-regulated protein 78 during oral cancer progression and the prognostic value in oral precancerous lesions. Head Neck.

[65] Chen YJ, Liao CT, Chen PJ, Lee LY, Li YC, Chen IH, Wang HM, Chang JT, Chen LJ, Yen TC, Tang CY, Cheng AJ (2011). Downregulation of Ches1 and other novel genes in oral cancer cells chronically exposed to areca nut extract. Head Neck.

[66] Chen YJ, Lee LY, Chao YK, Chang JT, Lu YC, Li HF, Chiu CC, Li YC, Li YL, Chiou JF, Cheng AJ (2013). DSG3 facilitates cancer cell growth and invasion through the DSG3- plakoglobin- TCF/LEF- Myc/cyclin D1/MMP signaling pathway. PloS one.

[67] Lee LY, Chen YJ, Lu YC, Liao CT, Chen IH, Chang JT, Huang YC, Chen WH, Huang CC, Tsai CY, Cheng AJ (2015). Fascin is a circulating tumor marker for head and neck cancer as determined by a proteomic analysis of interstitial fluid from the tumor microenvironment. Clin Chem Lab Med.

