# Supplementary material for: GDF15 contributes to radioresistance and cancer stemness of head and neck cancer by regulating cellular reactive oxygen species via a SMAD-associated signaling pathway
Source: Oncotarget. 2016 Nov 26;8(1):1508–28. doi: 10.18632/oncotarget.13649 (PMC5352073; doi:10.18632/oncotarget.13649)
Supplement: Supplementary file 1 [file oncotarget-08-1508-s001.pdf]

GDF15 contributes to radioresistance and cancer stemness of head and neck cancer by regulating cellular reactive oxygen species via a SMAD-associated signaling pathway

SUPPLEMENTARY TABLES

Supplementary Table S1: List of genes and primer sequences used in this study

Gene	Primer sequence
GDF15	5'- AGATCAAGACGAGCCTGCACC -3'
	5'- CATTCCACAGGGCAGGACAA -3'
CD44	5'- AGATCAGTCACAGACCTGCC -3'
	5'- GCAAACTGCAAGAATCAAAGCC -3'
b-actin	5'- CTCCTTAATGTCACGCACGATTTC-3'
	5'- GTGGGGCGCCCCAGGCACCA -3'

Supplementary Table S2: List of antibody sources used in this study

Antibody	Clone #	Company
GDF15/NAG-1	07-217	Millipore (Billerica, MA, USA)
CD44	555479	BD Biosciences (Franklin Lakes, NJ, USA)
CD44 isotype IgG	555743	BD Biosciences (Franklin Lakes, NJ, USA)
ALDH1	611194	BD Biosciences (Franklin Lakes, NJ, USA)
Nestin	sc-23927	Santa Cruz Biotech (Santa Cruz, CA, USA)
phosphor-SMAD sampler kit	9963s	Cell Signaling (Danver, MA, USA)
GAPDH	GT239	GeneTex (Irvine, CA, USA)
